# A high-resolution dataset of mouse brain vasculature for deep learning-based reconstruction

**DOI:** 10.3389/fninf.2026.1809341

**Published:** 2026-05-19

**Authors:** Xinwei Du, Shijun Li, Xiaojun Wang, Yuan Shen, Tingwei Quan

**Affiliations:** 1 North Alabama International College of Engineering and Technology, Guizhou University, Guiyang, Guizhou, China; 2 Key Laboratory of Animal Genetics, Breeding and Reproduction in the Plateau Mountainous Region, Ministry of Education, Guizhou University, Guiyang, Guizhou, China; 3 College of Animal Science, Guizhou University, Guiyang, Guizhou, China; 4 Key Laboratory of Biomedical Engineering of Hainan Province, School of Biomedical Engineering, Hainan University, Sanya, China; 5 Britton Chance Center for Biomedical Photonics, Wuhan National Laboratory for Optoelectronics, Huazhong University of Science and Technology, Wuhan, Hubei, China

**Keywords:** cerebrovascular imaging, neurovascular dataset, semi-automatic annotation tool, vascular network reconstruction, vessel skeletonization

## Abstract

Vascular network reconstruction is a crucial step in extracting vessel morphology and establishing its topological relationships from biological imaging data, holding significant scientific importance for studying brain structure and function, metabolism, and disease mechanisms. Current methods for vascular network reconstruction typically follow a “segment-first, then reconstruct” pipeline: first generating a binary segmentation from vascular images, followed by topological modeling. However, due to significant variations in vessel diameters, frequent presence of luminal voids in large vessels, and the complex, densely distributed nature of capillaries, existing approaches still face notable limitations in reconstruction accuracy. To address this, this study introduces and releases an annotated dataset of mouse brain vasculature. The dataset comprises 60 3D image blocks with the size of 512 × 512 × 512 acquired from four mouse brain samples using fluorescence micro-optical sectioning tomography (fMOST) imaging. It encompasses diverse structural morphologies ranging from large vessels to capillaries, with detailed annotations specifically targeting challenging vascular regions. Additionally, we provide a standardized vascular annotation pipeline and associated tools. This dataset aims to serve as a benchmark to support the development, evaluation, and comparison of algorithms for vascular network segmentation, reconstruction, and related tasks.

## 1 Introduction

The vascular network is a crucial transport and regulatory system in living organisms, and its topological structure and morphological features directly impact the function and health of tissues and organs (Hirsch et al., 2012; Holl et al., 2024; Zilles and Amunts, 2010). In the field of neuroscience, reconstructing and quantitatively analyzing the brain’s vascular network plays an indispensable role in revealing the connections between brain structure and function (Wälchli et al., 2015; Xiong et al., 2017), understanding neural metabolic mechanisms (Johnson, 2023; Kopylova et al., 2023), and exploring the pathological basis of cerebrovascular diseases (Girouard and Iadecola, 2006; Völgyi et al., 2018; Zhang et al., 2012). Vascular network reconstruction technology aims to automatically identify vessel voxels from microscopic imaging data and extract their geometric skeletons and topological connections (Moccia et al., 2018; Wälchli et al., 2021). This process transforms two- or three-dimensional images into graph structures suitable for quantitative analysis, serving as a key bridge connecting vascular imaging and quantitative analysis.

Currently, deep learning-based methods have become the mainstream technical approach for vascular segmentation, which is the critical first step for subsequent network reconstruction. For instance, the VesSAP method achieves whole-brain scale vascular network reconstruction from light-sheet fluorescence microscopy data by constructing a lightweight fully connected network combined with data augmentation techniques (Todorov et al., 2020). The TubeMap tool can support the reconstruction, visualization, and analysis of vascular networks in terabyte-scale datasets (Kirst et al., 2020). Li et al. (2023a) proposed a deep learning-based workflow for vascular segmentation and reconstruction, utilizing parallel computing techniques to achieve whole-brain mouse vascular network reconstruction at capillary resolution. In addition to these comprehensive frameworks, deep learning models tailored to the structural characteristics of vascular network have been proposed (Goni et al., 2022; Huang et al., 2018; Li et al., 2022, 2023b; Liu et al., 2024; Livne et al., 2019; Rougé et al., 2024; Wang et al., 2025; Yang et al., 2024). These include multi-scale feature fusion networks (Goni et al., 2022; Livne et al., 2019; Yang et al., 2024), loss functions designed to preserve topological continuity (Huang et al., 2018; Rougé et al., 2024), transformer-based long-range dependency modeling (Li et al., 2023b), and the introduction of novel architectures such as state-space models (Liu et al., 2024; Wang et al., 2025), all of which have further advanced methodological development in this field. While deep learning has achieved impressive performance in vascular image analysis, it still presents certain limitations. These include a strong dependence on human trainers and manual annotations, as well as potential unknown biases introduced by variations in experimental conditions, which can affect the reliability and generalization of learned models.

In addition to deep learning-based vascular image segmentation methods, the extraction of vascular centerlines is also a core component of vascular reconstruction. Typical skeletonization methods (Lee et al., 1994; Palágyi and Kuba, 1999; Zhang and Suen, 1984) operate on binary vascular images by iteratively removing boundary voxels that satisfy predefined constraints, gradually shrinking the target region to a medial axis that preserves topological connectivity. The accuracy of such methods depends on the quality of the binary image; moreover, they are prone to artifacts such as spurious branches and fractures caused by binarization errors, and are sensitive to vascular gaps and imaging noise. Model-driven methods characterize vascular morphology using primitives such as superellipsoids (Tyrrell et al., 2007), tubular structures (Goyal et al., 2012), and templates (Govyadinov et al., 2018), and achieve accurate extraction of vascular centerlines based on the central points of various models. Their key advantages include strong noise robustness and excellent representation capability for complex vascular geometries, although they suffer from high computational cost and are limited by computing resources in large-scale vascular image analysis. Notably, the method proposed by Mihelic et al. (2021) analyzes vascular morphological features and constructs a customized multi-scale filtering framework, which can directly extract and recover vascular centerlines and diameter information from complex raw vascular images. This method achieves segmentation-free direct vascular vectorization, which meets the requirements for large-scale image data processing (Mihelic et al., 2024).

For the evaluation metrics of vascular reconstruction, topological structure serves as a core metric for assessing reconstruction performance (Kirst et al., 2020; Moccia et al., 2018; Todorov et al., 2020; Wälchli et al., 2021). The key evaluation lies in the accuracy of three aspects: skeleton detection, branch point localization, and endpoint localization, all of which are highly correlated with the overall precision of topological reconstruction. On the basis of achieving accurate topological reconstruction, further quantitative analysis can be performed on details such as branching angles and graph structural features of the vascular network (Ji et al., 2021; Wu et al., 2014).

In three-dimensional optical imaging, the cerebral blood vessels exhibit high morphological complexity, posing challenges to the robustness and accuracy of vascular reconstruction algorithms. Specifically, vessel radii span several orders of magnitude, ranging from large vessels tens of micrometers in diameter to capillaries barely wide enough for a single red blood cell, all coexisting within the same field of view (Xiong et al., 2017). This demands algorithms with multi-scale perception capabilities. Larger vessels often exhibit internal voids in cross-section due to staining or imaging issues, leading to discontinuous local features. In capillary regions, dense structures and low contrast can cause adhesion or fragmentation between vessels (Angus and Wright, 2000; Kirst et al., 2020; Lang et al., 2012; Takahashi et al., 2022; Todorov et al., 2020). These factors cause existing algorithms to frequently produce fragmentation, false connections, or topological errors in predictions within complex regions, thereby compromising the accuracy of subsequent reconstructions. One limiting factor in the development of methods in this field is the lack of a publicly available dataset that systematically encompasses these challenging structures and provides high-precision annotations.

To address this challenge, we construct and release a high-quality, highly complex annotated dataset of the complete mouse cerebrovascular network. Acquired using fluorescence micro-optical sectioning tomography (Li et al., 2010; Wang et al., 2021), the dataset comprises 60 three-dimensional image blocks of size 512 × 512 × 512 from four mouse brains. This dataset encompasses diverse and challenging vascular morphologies from large vessels to dense capillaries, which is to support the development, evaluation, and benchmarking of algorithms for vascular image segmentation and 3D network reconstruction. To ensure labeling consistency and reproducibility, we propose a semi-automated vessel annotation pipeline and provide corresponding proofreading and quality control tools, thereby guaranteeing topological correctness in the annotation results. Furthermore, we conduct a benchmark evaluation of typical vascular segmentation algorithms on this dataset, quantitatively assessing their performance and limitations across varying vascular structures.

## 2 Materials and methods

### 2.1 Data collection

The image dataset used in this study was obtained from fluorescently labeled brains of four 8-weeks-old male C57BL/6 mice. The blood vessels were labeled via tail vein injection, following the procedure outlined below. First, all necessary reagents and instruments were prepared, including 0.01 M PBS, 4% PFA, 1% pentobarbital sodium, DyLight 594 dye, a constant-flow pump, dissection tools, and syringes. At the start of the experiment, the 0.01 M PBS was pre-warmed to 37 °C, while the DyLight 594 solution (1 mg/mL) and 4% PFA were pre-cooled to 4 °C.

The mouse was anesthetized (using gas anesthesia or intraperitoneal injection of 1% pentobarbital sodium), restrained, and then injected with 0.2 mL of DyLight 594 via the tail vein. After a 15-min waiting period, cardiac perfusion was performed. The heart was exposed, a needle was inserted into the left ventricle, and the right atrium was cut open. Perfusion was first carried out using 37 °C 0.01 M PBS until the liver turned pale, followed by pre-cooled 4% PFA for pre-fixation until the tail lifted and the body stiffened. The brain was then removed and placed in pre-cooled 4% PFA for 24 h at 4 °C in the dark, followed by washing with pre-cooled PBS and storage at 4 °C protected from light. All animal experiments were conducted in compliance with the protocols approved by the Animal Ethics Committee of Huazhong University of Science and Technology.

The dataset was acquired using fluorescence micro-optical sectioning tomography (fMOST; BioMapping 5000, OEBio Co., Ltd.). Continuous layer-scan imaging was performed with micron-level voxel resolution. In total, more than 5,000 coronal sections were collected, generating over 3.8 terabytes of uncompressed image data (8-bit pixel depth). From the four whole-brain datasets, we selected a total of 60 image blocks with dimensions of 512 × 512 × 512 voxels for annotation. The main processing steps were as follows: (i) the original image slices were downsampled so that each voxel corresponded to a physical size of 1 μm × 1 μm × 2 μm. (ii) Using BVLab software, the data were converted to a pyramid-style big-data format for block-wise browsing within BVLab. Here, BVLab is simply a more concise name for the BioimageVision software (Cai et al., 2025); (iii) data blocks corresponding to major vessel pathways were identified by browsing along large vessels in BVLab and exported. (iv) Finally, by globally browsing the whole-brain data in BVLab, we specifically selected data blocks containing capillary regions (without large vessels).

To reduce GPU memory constraints during deep learning training, each 512 × 512 × 512 block was evenly divided into 1,620 smaller blocks of size 196 × 196 × 196, with overlapping boundaries between adjacent blocks to preserve spatial continuity. Based on the gray-level statistical distribution of these 1,620 blocks, K-means clustering was applied to group them into 50 clusters, from which 199 representative blocks were randomly sampled to form the training set. This training set was used to train the segmentation network, which helps reduce errors in the semi-automatic annotation procedure and improves the accuracy of vessel reconstruction in automatic analysis. Meanwhile, 108 independent blocks were selected to support the experimental results of this study.

### 2.2 Human-in-the-loop data annotation

**FIGURE 1:**
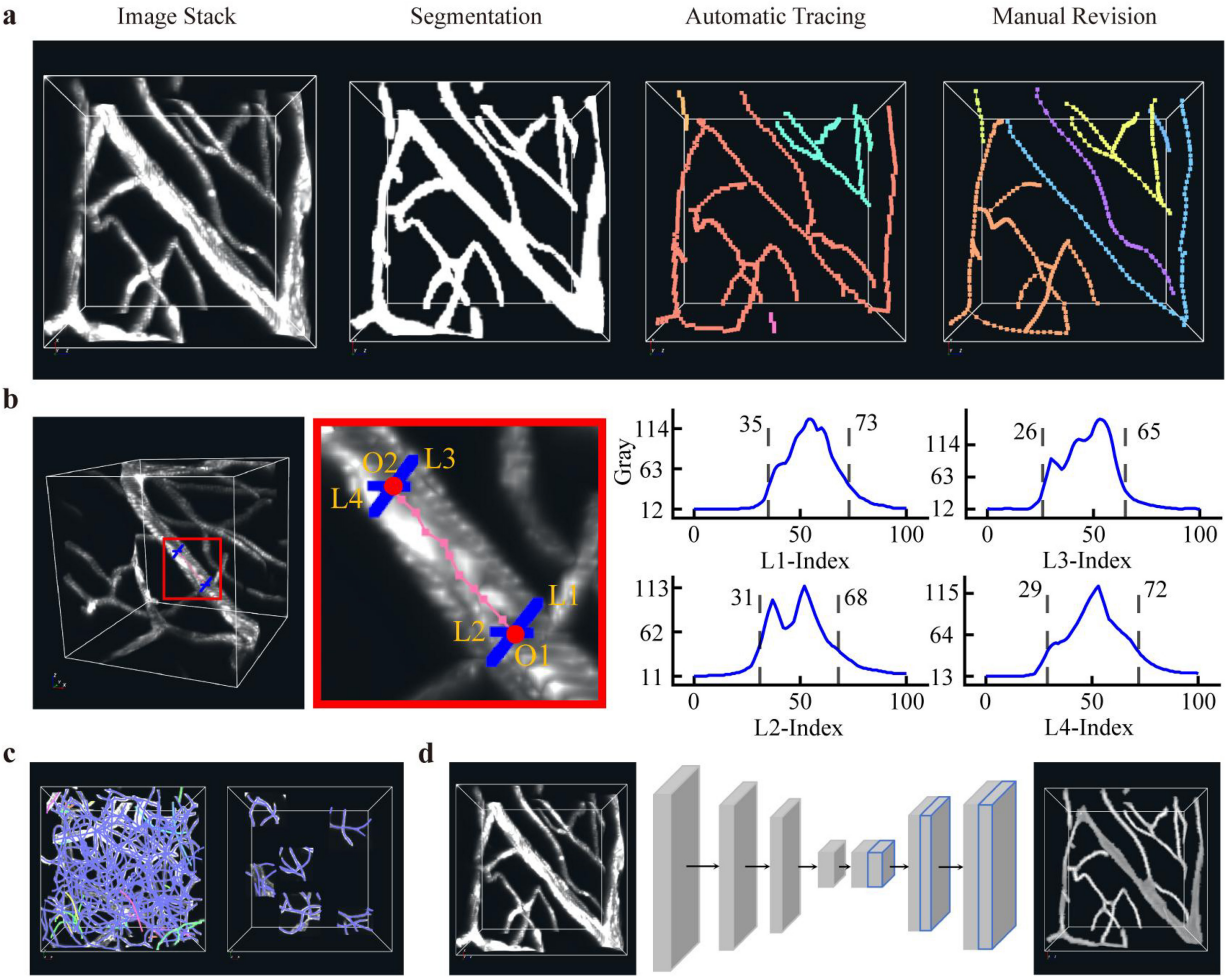
The pipeline for the rapid generation of a vascular annotation set. **(a)** The process from vascular imaging data to reconstruction results comprises three key steps: vascular segmentation, automatic skeleton tracing, and manual revision. **(b)** During manual revision, the diameter of large vessels is measured using a dual visual ray method. **(c)** To facilitate the correction of vascular skeleton connections, potential connection regions are selectively visualized, thereby minimizing interference from extraneous signals. **(d)** A U-Net network is trained to predict vascular images, producing a probability map of foreground vascular regions; this map is then thresholded to obtain the final vascular segmentation.

To enhance the efficiency of data annotation, we adopted a human-in-the-loop annotation strategy (Figure 1a). The overall workflow consists of four main steps. (i) Semi-Annotation: the skeleton and radii of blood vessels were traced via semi-manual operation, and binary vessel masks were generated through computation based on these annotations. (ii) Model Training: the manually annotated data were used to train a 3D U-Net network (Çiçek et al., 2016), resulting in a segmentation model capable of identifying vascular structures. (iii) Inference and Reconstruction: the trained model was applied to the remaining unlabeled data to predict vessel masks. Subsequently, the voxel scooping technique (Huang et al., 2021; Rodriguez et al., 2009) was employed on the predicted masks to automatically reconstruct vascular skeleton and their topological connections. (iv) Correction and Refinement: the automatically generated vessel skeletons were carefully revised, correcting missing connections, topological errors, and inaccuracies in tracing. This iterative approach combines manual expertise with automated model inference to achieve efficient and high-quality vascular annotation.

In the preliminary semi-automatic annotation stage, we generated vascular skeletons and radii through semi-automatic interactive tracing using the BVLab-Annotation tool, without any deep learning segmentation. Capillary radii were calculated from the skeleton information using an optimization-based method (Li et al., 2020) and manually checked for accuracy. Deep learning segmentation is not involved in the preliminary semi-automatic annotation step, and it is only introduced in the later inference and reconstruction stage to boost the accuracy of vascular skeletonization.

During our semi-automatic reconstruction pipeline of vascular images, the challenges include accurately identifying vessel diameters and correcting their topological connections. To address this, we proposed the following approach and extended BVLab software to develop the BVLab-annotation module to accelerate the labeling process. This module is designed for semi-automatic annotation and topological correction of vascular images. It supports the import of vascular images, SWC skeleton files and image folders, with core functions including revision of vascular topological structures and semi-automatic radius annotation. It enables manual correction of branch points and endpoints, and features built-in shortcut operations, which can effectively improve the efficiency of annotation and revision, reduce manual intervention costs, and provide convenient tool support for relevant researches.

The 3D vascular images visualized in the annotation software are essentially 2D projections of volumetric data along a given viewing direction. As a result, it is not possible to precisely determine the true 3D spatial position of a vessel solely from a single projection plane. To enable accurate spatial localization and diameter annotation, we have designed an interactive dual-view ray intersection and signal profile analysis method (Figure 1b). The specific workflow is described as follows:

a) Dual-View Ray Intersection for 3D Positioning: in the first view (projection plane A), the user manually selects the center of the target vessel segment in the 2D projected image. The system records the corresponding screen coordinates and, based on the current camera parameters, reconstructs a 3D ray R_1_ originating from the viewpoint and passing through that pixel. After rotating or shifting the 3D image to a distinctly different second view (projection plane B), the user again clicks on the corresponding location of the same vessel segment. The system then generates a second 3D ray R_2_. By computing the closest point between rays R_1_ and R_2_ (i.e., the midpoint of their common perpendicular segment), the system determines the 3D spatial location of the vessel center point P_*c*_. Additionally, the system calculates the projection points P_1_ and P_2_ of P_*c*_ onto R_1_ and R_2_, respectively, for further analysis.

b) Signal Intensity Profile along the Rays: centered at projection points P_1_ and P_2_, the system samples image intensity values (e.g., grayscale values) along the ray direction and its opposite direction at unit voxel intervals. A dynamic curve plotting “sampling position vs. signal intensity” is displayed in the software interface, reflecting how the signal varies across the vessel along each ray.

c) Interactive Interval Selection and Radius Estimation: the operator examines the intensity profile and, based on typical vascular signal characteristics in the image, manually selects a continuous interval on the curve that corresponds to the signal segment between the vessel walls. The length of this selected interval is then used to estimate the vessel radius according to the following formula (Equation 1):


$$\text{r}=0.25\,\left({\text{L}}_{1}\,\sqrt{{\left({\text{d}}_{1}\,\text{s}\right)}_{\text{x}}^{2}+{\left({\text{d}}_{1}\,\text{s}\right)}_{\text{y}}^{2}+{\left({\text{d}}_{1}\,\text{s}\right)}_{\text{z}}^{2}}+{\text{L}}_{2}\,\sqrt{{\left({\text{d}}_{2}\,\text{s}\right)}_{\text{x}}^{2}+{\left({\text{d}}_{2}\,\text{s}\right)}_{\text{y}}^{2}+{\left({\text{d}}_{2}\,\text{s}\right)}_{\text{z}}^{2}}\right)$$ (1)

Here, L_1_ and L_2_ represent the lengths of the selected intervals on rays R_1_ and R_2_, respectively, and *s* denotes the voxel size (1 μm in the x and y directions, and 2 μm in the z direction). d_1_ and d_2_ are the directional vectors of rays R_1_ and R_2_. The subscripts x, y, and z inside the square root indicate the component-wise multiplication of the two vectors.

It is worth noting that our capillary diameter annotation involved an additional quality control step: after skeleton extraction and optimization-based diameter estimation (Li et al., 2020), we manually inspected and corrected regions exhibiting abrupt diameter variations along the skeleton.

Correcting vascular connectivity is a critical step for accurately determining topological structure, which is essential for subsequent analyses such as calculating inter-vessel angles and localizing branching points. However, manually correcting connections in 3D is often time-consuming because vessels frequently occlude one another in standard visualizations, making their relationships difficult to discern clearly.

To address this, we introduce a SmartVision strategy (Zhou et al., 2021), which shifts the annotator’s focus from the entire data volume to local regions of high uncertainty, thereby significantly improving the efficiency of connectivity correction (Figure 1c). This strategy automatically identifies potential branching locations where connectivity may be unclear, such as regions containing two or more endpoints of reconstructed vessel segments, or one or more previously identified branch points within a local image range. It then extracts the corresponding local image signals and reconstructed vessel segments. For branch points that are straightforward to verify, the user can simply mark them as “checked.” In more complex or ambiguous cases, the tool provides interactive, multi-view 3D visualization of the local region while suppressing signals from surrounding areas, allowing the user to inspect and correct connectivity relationships more intuitively and accurately.

After topological correction, we fix the skeleton endpoints and branch points, then apply a hybrid smoothing strategy combining moving average and B-spline interpolation to enhance smoothness while preserving topology. Using the corrected skeleton, we estimate the corresponding vessel radii as described above. The skeleton is then uniformly sampled at one voxel intervals, and a Gaussian kernel with width matched to the local vessel radius is applied at each point. Large vessel radii are annotated manually, while capillary radii are computed algorithmically and manually refined. This procedure yields the final vascular morphology annotation map to supervise the segmentation network.

In our semi-automated pipeline, annotating a 196 × 196 × 196 voxel block takes 20 min on average, representing a substantial efficiency gain over manual annotation, which requires at least 20 min per block to annotate thick vessel morphology alone.

### 2.3 Network training

We employ a 3D U-Net architecture to train a vascular network segmentation model using the annotated data (Figure 1d). The network follows the standard 3D U-Net structure. In the encoder, four down-sampling stages are used, each consisting of two 3 × 3 × 3 convolutional kernels followed by ReLU activation and batch normalization. Down-sampling is performed via 2 × 2 × 2 max-pooling, with the number of feature channels doubling from 16 to 512. In the decoder, transposed convolutions are used for up-sampling to restore the feature map resolution to the original size. After each up-sampling step, the feature maps are concatenated with the corresponding feature maps from the encoder via skip connections, enabling multi-scale feature fusion. The output layer uses a 1 × 1 × 1 convolutional kernel and a sigmoid activation function to produce a probability map indicating whether each voxel belongs to a vessel.

All experiments were conducted on an NVIDIA GeForce GTX 1080 Ti GPU using the PyTorch 2.1 framework. The model was optimized with the Adam optimizer, with an initial learning rate of 2 × 10^–4^ and a batch size of 1.

The training dataset consists of 199 manually annotated 3D vascular image blocks, each of size 192 × 192 × 192 voxels, covering diverse vessel radii and vascular densities. The dataset was split into 139 blocks for training, 40 for validation, 20 for testing. A combined loss function L_1_ (Li et al., 2025) was used during training (Equation 2).


$$\mathrm{Loss}\,\left({\text{y}}_{\text{p}},{\text{y}}_{\text{g}}\right)=1/N\,{||{\text{y}}_{\text{p}}-{\text{y}}_{\text{g}}||}_{1}+1/\#\,{\text{S}}_{1}\,{||{\left({\text{y}}_{\text{p}}-{\text{y}}_{\text{g}}\right)}_{{\text{S}}_{1}}||}_{1}$$ (2)

$$+1/\#\,{\text{S}}_{2}\,{||{\left({\text{y}}_{\text{p}}-{\text{y}}_{\text{g}}\right)}_{{\text{S}}_{2}}||}_{1}+1/\#\,S_{2}^{*}\,{||{\left(y_{p}-y_{g}\right)}_{S_{2}^{*}}||}_{1}$$

Here, *y_p_* and*y_g_* represent the segmentation network output and the ground truth, respectively. *S_1_* and *S_2_* are the regions of the ground truth image where the voxel intensity exceeds 0.012 and 0.404 (normalized intensity), respectively. ${}_{2}^{*}$ is the region of the segmentation output in which the voxel intensity is more than 0.404. _#_ denotes the total number of voxels in the region.

The design of the combined loss function is closely related to the generation strategy of the foreground regions, which are derived from the semi-automatically extracted skeleton and corresponding radius to construct the foreground probability map. Specifically, *S*_1_ denotes the strict foreground region, *S*_2_ includes the transition zone between the foreground and background, and *S*_2_* represents the foreground region predicted by the network. The total loss comprises four components, where the first three loss terms enforce the segmentation regions to be consistent with the semi-automatically annotated foreground, while only the first term aligns the network-predicted background with the semi-automatic foreground annotation, essentially forming a weighted constraint mechanism. In particular, the loss associated with the *S*_2_* region is introduced to further suppress false-positive segmentation and improve the prediction accuracy of the network.

### 2.4 Baseline tracing methods

Vessel segmentation networks often produce hollow structures in tubular vessels, and this case requires filling for subsequent tracing. The processing pipeline is as follows: first, apply nearest-neighbor downsampling to the segmented image, reducing its size to half of the original. Normalize the downsampled image by mapping voxel values to the range [0, 1], followed by smoothing with Gaussian filtering (typically using a 3 × 3 kernel with a standard deviation of 2). Perform binarization using the Otsu method (Liu and Yu, 2009) and fill internal cavities with morphological closing operations. Finally, upsample the processed binary image to the original resolution via interpolation and merge it with the initial segmentation using a logical OR operation, preserving regions marked as foreground in either image. The merged segmentation effectively fills internal hollow regions while retaining the boundary and contour information of the original result. This provides a structurally complete and well-connected geometric foundation for subsequent skeleton extraction.

Based on this, three representative baseline algorithms were selected for vessel skeleton tracing: The thinning method (Lee et al., 1994): a classical skeletonization algorithm for binary images. It iteratively removes boundary pixels to produce a one-pixel-wide centerline while preserving topology. Voxel scooping method (Ji et al., 2021): a geometry-based centerline extraction algorithm in 3D space. Its core idea is to refine the position of each skeleton point by fitting a sphere within its local neighborhood and determining the propagation direction along the vessel. VesselVio (Bumgarner and Nelson, 2022) is an open-source Python package dedicated to vascular network analysis, offering a complete workflow from visualization and skeleton extraction to morphological quantification. In this study, its built-in vessel tracing module is used as a baseline method to compare the performance of different algorithms on the same data.

These three methods represent classical technical approaches to vessel skeleton extraction from morphological, geometric optimization, and integrated tool perspectives. To ensure a fair comparison, all algorithms were run on the same preprocessed segmentation maps. This segmentation-based reconstruction paradigm, with deep learning used exclusively for vessel segmentation followed by conventional skeletonization, is consistent with state-of-the-art workflows such as VesSAP (Todorov et al., 2020) and TubeMap (Kirst et al., 2020). End-to-end deep learning reconstruction methods remain beyond the scope of this data report.

### 2.5 Evaluation metrics

We adopt a centerline intersection-based F1 score, which shifts the evaluation focus from voxel overlap to centerline accuracy. The reconstructed skeleton consists of connected points with sub-voxel accuracy and is stored in the standard SWC format. The custom F1-score measures the matching degree between the automatically reconstructed skeleton and the semi-automatically generated ground truth, an evaluation protocol widely adopted in both neuron and vascular reconstruction. By assessing consistency at the centerline level, we can more directly reflect the quality of structural connectivity in the segmented vascular networks. The F1-score calculation process is as follows.

We call the skeleton produced by our method P (for Predicted), and the skeleton from the human annotations G (for Ground Truth). To evaluate how well P matches G, we calculate three standard metrics: recall, precision, and the F1 Score. To compute these metrics, we first define a matching rule, shown in Equation 3:


$$\text{I}(\text{p},\text{G})\;=\;\left\{ \begin{array}{ll} \text{1}, & \min_{\text{q}\in \text{G}}{\text{||p-q||}}_{\text{2}}<\text{Thre}\\ 0, & \text{otherwise} \end{array}\right.$$ (3)

Here, | | | | _2_ stands for the squared 2-norm, and *Thre* is a predefined threshold set to 3 for evaluating skeleton tracing and 5 for evaluating branch point localization. A point *p* in the predicted set P is considered a correct match if the distance to its nearest point *q* in the manually labeled set G is less than or equal to *Thre*. The thresholds of 3 and 5 voxels correspond to physical distances of 3–6 μm and 5–10 μm, respectively, given the voxel size of 1 × 1 × 2 μm. These values are determined as a trade-off to balance the F1-score and topological accuracy: a smaller threshold leads to an overly low F1-score even for correct topology, while a larger threshold inflates the score but introduces false structures. Based on this matching rule, we calculate recall (Equation 4), precision (Equation 5), and F1-Score (Equation 6) using the formulas below.


$$\mathrm{Recall}\,\left(\text{P},\text{G}\right)=\frac{\sum_{\text{g}\in \text{G}}\text{I}\,\left(\text{g},\text{P}\right)}{{\text{N}}_{\text{g}}}$$ (4)

$$\mathrm{Precision}\,\left(\text{P},\text{G}\right)=\frac{\sum_{\text{n}\in \text{P}}\text{I}\,\left({\text{p}}_{\text{i}},\text{G}\right)}{{\text{N}}_{\text{P}}}$$ (5)

$$\mathrm{F1}-\mathrm{Score}=2\,\frac{\mathrm{Recall}\times \mathrm{Precision}}{\mathrm{Recall}+\mathrm{Precision}}$$ (6)

Here N_g_ denotes the number of points in the ground truth set generated from manual annotations, and N_p_ represents the number of points in the set generated by the predicted segmentation.

We abstract the reconstructed vascular network as a graph structure: the bifurcation and endpoints of the skeleton are regarded as graph nodes, and a graph edge is defined as a continuous skeleton segment connecting a pair of adjacent nodes (two bifurcation points, or one bifurcation point and one endpoint). The node degree is defined as the number of graph edges connected to each node. These graph-based statistical metrics are also applied in our reconstruction.

## 3 Results

### 3.1 Reliability analysis of data annotation pipeline

We analyze the reliability of the entire annotation pipeline. As elaborated above, our pipeline employs automatic techniques to improve annotation efficiency: a segmentation network is used to enhance image signals and improve skeletonization accuracy, and an optimization-based model is applied to estimate capillary radii. From the semi-automatically annotated skeletons and radii, we generate probability maps that serve as supervision for network training. On one hand, we verify whether these supervision probability maps can effectively guide the segmentation network to produce reliable predictions, and whether the automatically extracted skeletons are consistent with the semi-automatically annotated references. On the other hand, we evaluate the inter-annotator variability under the same automatic pipeline, so as to quantify the influence of human subjectivity on annotation consistency.

For evaluation, 108 independent image blocks were selected to support all experiments in this study, with half sampled from the training set and the other half from data outside the training set. Since our semi-automatic ground-truth is generated from skeleton and radius estimation, where segmentation serves only as an auxiliary step to reduce reconstruction errors rather than providing an independent manual label, this evaluation strategy is reasonable for validating the proposed reconstruction method.

**FIGURE 2:**
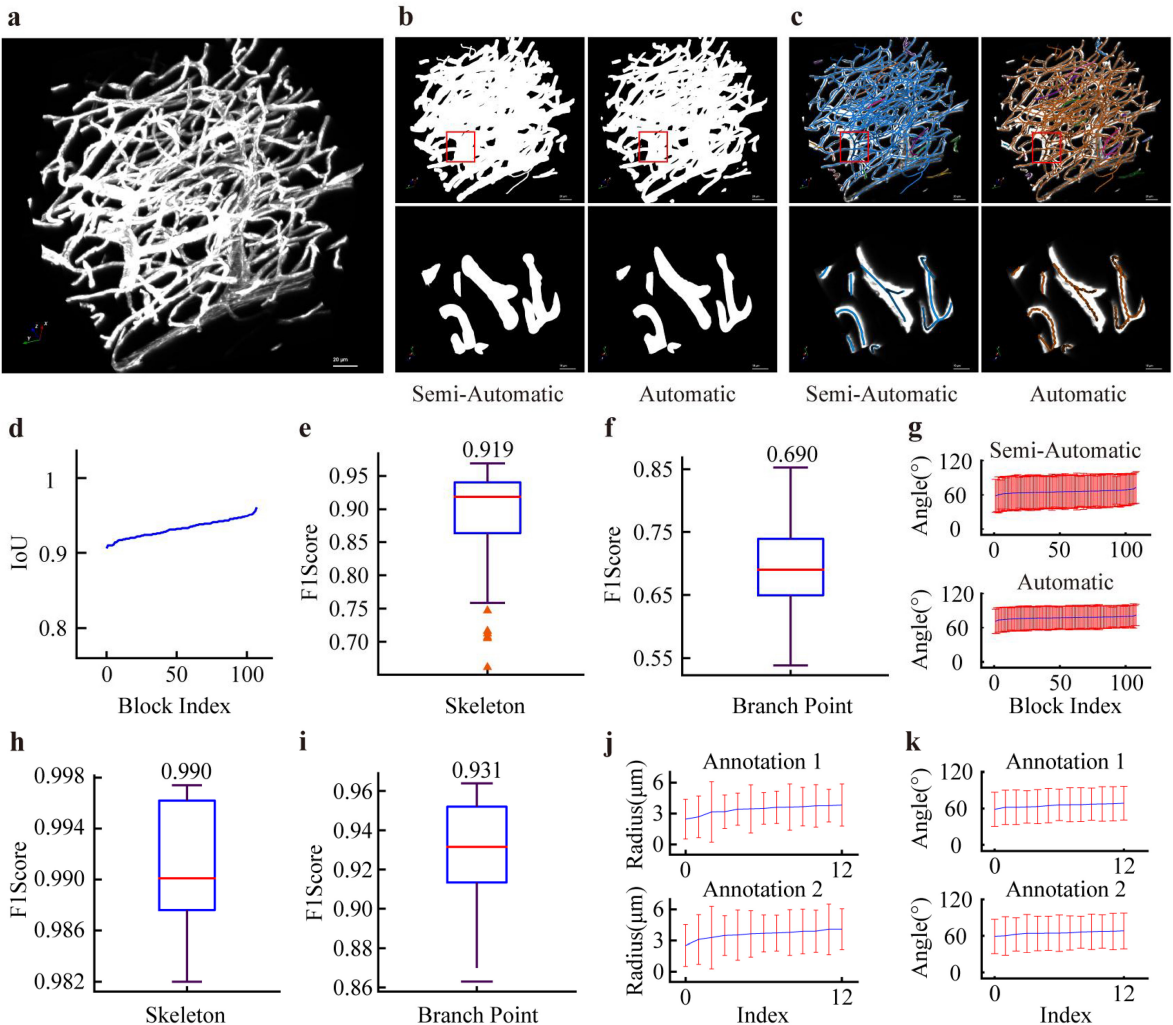
Illustrates the reliability validation of the annotated data. **(a)** An initial data block. **(b)** Comparison between the semi-automatic segmentation mask generated from our skeleton and radius annotation pipeline and the deep learning-based automatic segmentation mask predicted by U-Net, which was trained using probability maps derived from semi-automatic annotations as ground-truth. **(c)** Skeletons obtained by our semi-automatic pipeline and those from the voxel scooping method, whose automatic tracing procedure is described in the section “2.4 Baseline tracing methods,” are also compared. **(d)** Quantitative consistency analysis between semi-automatic and deep learning-based automatic segmentation. For 108 data blocks, quantitative consistency between semi-automatic labeling and results from the voxel scooping method is further assessed in terms of skeleton extraction **(e)**, branch point localization **(f)**, and branch point angles **(g)**. Additionally, for 12 distinct data blocks, the consistency between two annotators within the semi-automated pipeline is evaluated across extracted skeleton **(h)**, branch point localization **(i)**, vessel diameter **(j)**, and branch angles **(k)**.

Figures 2a–c present representative examples that visually verify our supervision probability maps can effectively guide the segmentation network to produce reliable predictions and ensure consistency between automatically extracted skeletons and the semi-automatically annotated references. We further provide quantitative analysis to support these observations. The Intersection over Union (IoU) between automatic and semi-automatic segmentation exceeds 0.90, indicating high segmentation consistency (Figure 2d). For skeleton extraction, the F1-score between automatic and manual results reaches 0.92 (Figure 2e), demonstrating reliable skeleton detection. However, the F1-score for branch point localization is 0.69 (Figure 2f), which is relatively lower mainly due to: (1) numerous false branches in thick vessel regions; (2) deviations in the automatic tracing algorithm during branch point detection. Notably, the semi-automatic method yields significantly larger variation in branch angles than the automatic methods and provides a more faithful representation of their true distribution (Figure 2g).

Two independent annotators each labeled 12 volumes of size 192 × 192 × 192 voxels. Comparison of the extracted skeletons (Figure 2h) and branch points (Figure 2i) showed high consistency between the two annotations. Further statistical analysis of vessel diameter (Figure 2j) and branch angle (Figure 2k) derived from the labeled data also revealed minimal inter-annotator differences. Within our annotation pipeline, even with the involvement of automatic algorithms, the inter-annotator consistency is substantially higher than that achieved by fully automatic methods alone, demonstrating that automatic algorithms serve only as an effective aid, while human annotation plays the decisive role in ensuring high-quality labeling.

### 3.2 Analysis of data characteristics

**FIGURE 3:**
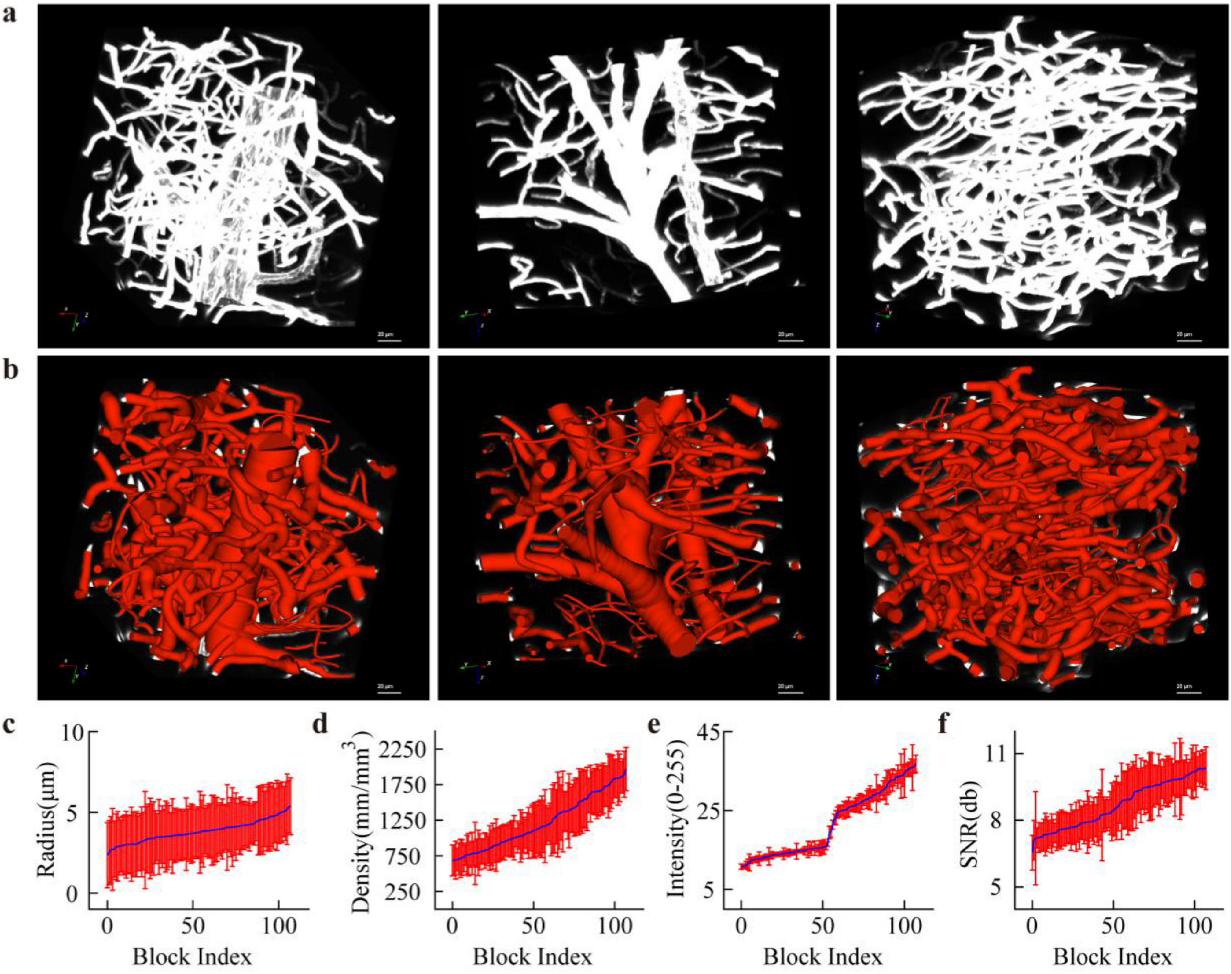
The analysis of data characteristics: **(a)** shows example data including thick vessels, medium vessels, and capillaries; **(b)** displays the reconstructed vascular network with skeleton structure and radius information using the semi-automatic method; **(c)** provides the vessel radius calculated at skeleton points by the same method, along with the average and standard deviation for each data block; **(d)** summarizes the vascular density in each block; **(e,f)** give the statistical results of signal strength and signal-to-noise ratio, respectively.

We present the characteristics of the annotated data. From the given 108 data blocks, we selected representative datasets and their reconstruction for illustration, including data containing extremely thick vessels, relatively thick vessels, and capillaries (Figures 3a,b). During presentation, we appropriately adjusted the dynamic range of the data to ensure vessels with weak signals remain clearly visible. We also display the diameter information of the annotated vessels, with these diameter measurements fully covering the signal regions of the vessels.

We computed four quantitative metrics (Figures 3c–f): vessel radius, vessel density, signal intensity, and signal-to-noise ratio (SNR). In calculating vessel radius and signal intensity, we performed statistics based on vessel skeleton points: within each data block, we extracted the radius values and signal intensities corresponding to the skeleton points, then calculated the mean and standard deviation of these parameters (Figures 3c,d). The diameter statistics show that although the average vessel diameter across data blocks varies smoothly, the variance is large, indicating the presence of some relatively thick vessels in the data. From the signal intensity perspective, the data includes extremely weak signals–some entire blocks exhibit overall low signal levels, with averages as low as 10.

Additionally, we calculated vessel distribution density (Figure 3e). The specific method is: each data block is divided into 27 sub-blocks of size 64 × 64 × 64; the total vessel length in each sub-block is computed and divided by its volume to obtain a local density value; statistics are then performed on these sub-block densities to derive the average density and its variance, reflecting the distribution of vessels within the data block. The results show significant variation in vessel density across different regions–in areas particularly dense with capillaries, the density can reach up to 2000 mm/mmł. We also calculated the SNR of the images by dividing the signal intensity by the background standard deviation (Figure 3f). The results indicate that although the overall image SNR is relatively high, some regions still exhibit weak signals.

This dataset exhibits good diversity and representativeness across vessel scale, signal intensity, and spatial distribution, supporting subsequent quantitative vascular analysis and model training. Moreover, the inclusion of weak-signal and high-density regions provides critical evidence for evaluating algorithm robustness.

### 3.3 Benchmark analysis of skeletonization algorithms

**FIGURE 4:**
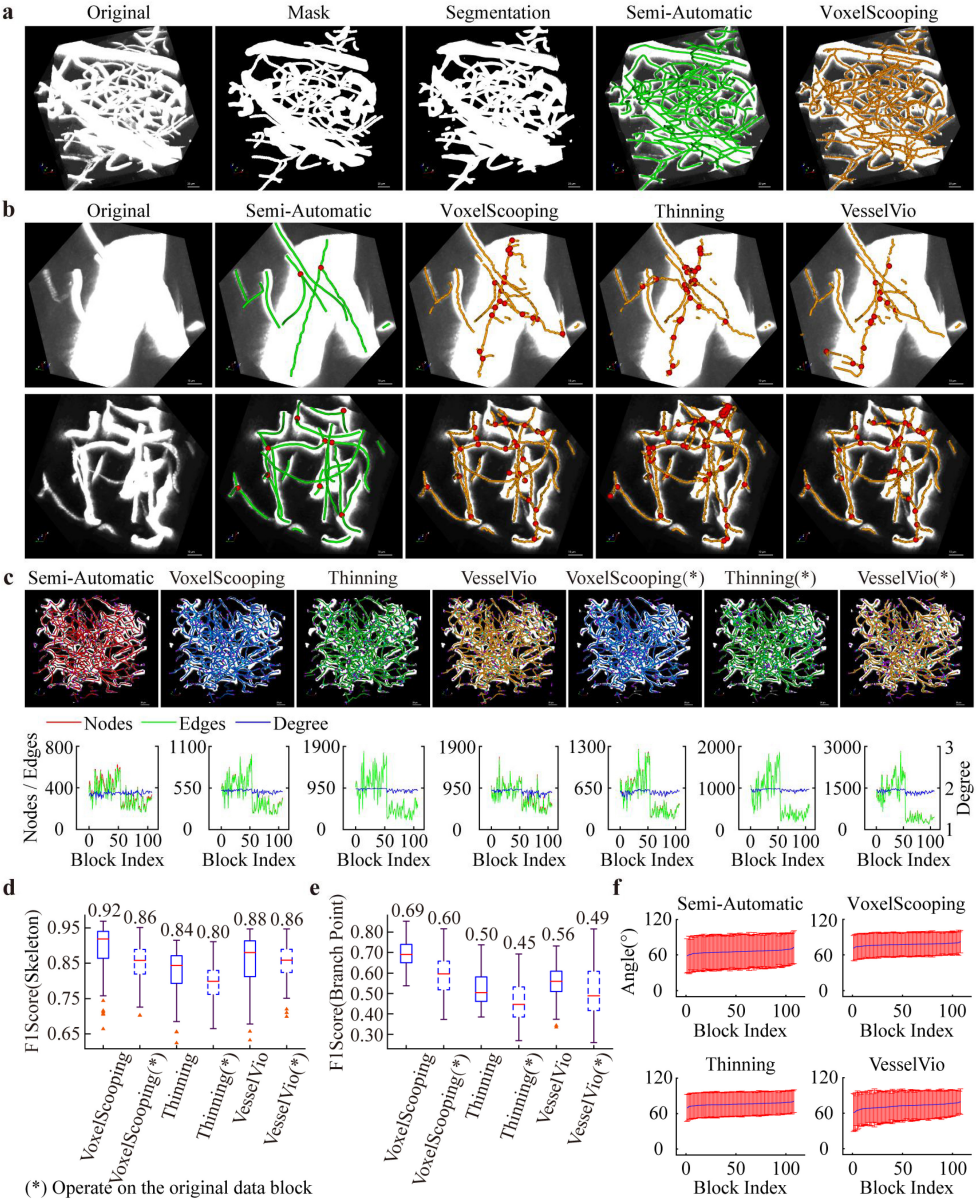
Analysis of annotated data using the baseline algorithm. **(a)** Example reconstructions from annotated data, including segmentation results and topology construction. **(b)** Typical reconstruction results comparing semi-automatic and fully automatic methods. The semi-automatic method follows our human-in-the-loop pipeline, which serves as the reference standard for evaluating fully automatic approaches. **(c)** Quantification of vascular reconstruction networks based on graph structure. **(d)** For the reconstructed skeleton, the automatic method performs reconstruction on both the segmented image and the raw data, and compares the results with the reference standard to calculate the F1-Score. **(e)** Branch points are extracted from the reconstruction and compared with the gold standard. **(f)** Branch angles are computed based on the reconstruction results.

We tested several classical skeletonization algorithms on the annotated dataset to verify their performance and set a comparable benchmark for our dataset. Using segmentation results generated by the network trained on our semi-automatic annotations, we applied these skeletonization algorithms to reconstruct blood vessels from the segmented masks. Figures 4a,b show the original images, corresponding vessel segmentation outputs, and vessel tracing results of representative sub-blocks, which cover vascular structures at various scales. Visual inspection reveals three prominent defects: first, numerous false skeletons and redundant branch points appear during skeleton extraction for thick vessels; second, obvious localization deviations exist at branch points (Figure 4b). These issues are mainly attributed to the high threshold sensitivity of mainstream skeletonization methods, which results in poor robustness when processing multi-scale vascular structures and makes the algorithms vulnerable to minor protrusions in the segmentation outputs.

To further quantify these visual observations and validate the above findings, we conducted a set of quantitative evaluations. Firstly, we abstracted the reconstructed skeleton network into a graph structure, where nodes represent vessel endpoints or branch points, and edges correspond to individual skeleton segments (Figure 4c). We counted the number of nodes and edges for each data block, and the maximum gap in node count reached up to 100% (one fold). The results present large topological differences between the outputs of automatic algorithms and those of our pipeline, obvious variations among different automatic methods, as well as notable discrepancies between results obtained from segmentation masks and raw images. These quantitative findings are well consistent with the visual observations shown in Figure 4b.

Secondly, we calculated the F1-score to assess the accuracy of skeleton extraction. Among all tested methods, the voxel scooping method achieved the best performance, with an average F1-score of 0.92. The other two methods also obtained desirable F1-scores of 0.84 and 0.88, respectively, suggesting that the extracted skeletons are highly consistent with the ground truth in most regions, except for areas with thick vessels (Figure 4d).

Thirdly, we evaluated the matching accuracy between automatically detected branch points and semi-automatic annotated landmarks. In sharp contrast to the high accuracy of skeleton extraction, the accuracy of automated branch point localization is considerably lower, with an average score below 0.7 (Figure 4e). This result fully proves that reconstructing complete and accurate vascular topology is far more challenging than extracting independent vessel skeletons alone.

To confirm the necessity of high-quality segmentation, we carried out additional control experiments: when these skeletonization algorithms were directly applied to raw images instead of our refined segmentation masks, the overall reconstruction accuracy dropped sharply (Figures 4d,e). In addition to the core quantitative metrics, we also analyzed the angular distribution of vessel segments in each data block. The results indicate that outputs from VesselVio are closer to semi-automatic annotations (Figure 4f), while the Voxel Scoping and 3D skeleton methods tend to produce larger angular deviations. Overall, although vessel skeleton extraction can achieve high overall accuracy with an F1-score up to 0.92, reconstructing fine-grained vascular topological structures remains highly difficult, as reflected by the low branch point localization accuracy (average score below 0.7). Therefore, future research should focus on algorithm optimization to improve the integrity and robustness of vascular network reconstruction.

## 4 Discussion and conclusion

We present a high-resolution annotated dataset of mouse cerebral blood vessels, which provides high-quality and diverse benchmark data for deep learning-based vascular network segmentation and reconstruction algorithms. Through systematic data acquisition, a semi-automated annotation pipeline, and detailed quantitative analysis, this dataset exhibits the following distinctive features and holds academic value.

The dataset encompasses multi-scale vascular structures, with vessel radii ranging from approximately 2 μm (capillaries) to 20 μm (large trunk vessels), and specifically incorporates anatomically challenging regions, including densely packed capillary networks and large vessels exhibiting complex morphology. The annotation procedure adopts a human-in-the-loop semi-automatic strategy equipped with specialized quality control tools, which effectively guarantees the topological correctness and geometric accuracy of the annotations. Quantitative evaluation presented in Figure 2 further validates the repeatability and reliability of the dataset annotations.

In addition to providing raw images and corresponding annotations, the dataset also contains standardized preprocessing pipelines and dedicated evaluation metrics. Benchmark tests performed on this dataset reveal that current classical skeleton extraction methods achieve relatively high overall extraction accuracy, with F1 scores reaching up to 0.92. Nevertheless, substantial limitations persist in branch point localization and topological connection reconstruction, as the relevant accuracy values generally remain below 0.7. This finding underscores that topological reconstruction of vascular networks still constitutes a critical challenge for existing algorithms.

This study has several notable limitations. First, the dataset is currently constructed using only four mouse brain samples. Although it covers diverse types of vascular architectures, its representativeness in terms of biological variability and region-specific anatomical features remains insufficient. Second, even though the annotation workflow is semi-automated, final verification of annotations in complex vascular regions still relies on manual inspection, which may introduce a certain degree of subjective bias. Third, the existing benchmark tests are only conducted on several classic skeleton extraction methods, without covering the latest deep learning-based reconstruction models such as Transformer-based networks and graph neural networks.

To address the drawbacks of traditional algorithms in branch point localization and topological connection reconstruction, as well as remedy the limitations of this work, several promising directions are proposed for future research. For targeted and immediate improvements of current algorithms, feasible strategies include: (1) refining segmented thick vessels with morphological information such as distance fields derived from vessel segmentation results, thereby suppressing reconstruction errors in large vessel regions; (2) designing novel skeleton tracing methods based on graph theory or global optimization frameworks to enhance the accuracy of topological connections; (3) developing intelligent post-processing systems that can automatically detect and correct topological defects.

Beyond the optimization of existing algorithms, vectorize-first strategies remain a crucial research direction for vascular network reconstruction (Govyadinov et al., 2018; Goyal et al., 2013; Mihelic et al., 2021; Tyrrell et al., 2007). In particular, recent studies have demonstrated that constructing tailored filters targeting vascular morphological characteristics can effectively enhance the image intensity of vascular central regions and reduce the difficulty of centerline extraction (Mihelic et al., 2024). Developing user-friendly software with visual programming functions, which enables researchers to freely combine and customize such filters, will further expand the applicability of image processing approaches to wider vascular scales and practical scenarios. Another pivotal direction is to improve branch point localization by introducing confidence estimation for branch point detection. Such a design allows automatic screening of unreliable detection results, leaving only low-confidence branch points for manual revision, which can drastically boost reconstruction efficiency and reduce manual labor costs.

## Data Availability

The datasets presented in this study can be found in online repositories. The names of the repository/repositories and accession number(s) can be found in the article/supplementary material.
